# Testicular cancer in Geneva, Switzerland, 1970–2012: incidence trends, survival and risk of second cancer

**DOI:** 10.1186/s12894-019-0494-0

**Published:** 2019-07-10

**Authors:** Robin Schaffar, Samaksha Pant, Christine Bouchardy, Hyma Schubert, Elisabetta Rapiti

**Affiliations:** Geneva Cancer Registry, Global Health Institute, University of Geneva, Geneva, Switzerland

**Keywords:** Testicular cancer, Second cancer, Trends, Incidence, Survival, Socio-economic status

## Abstract

**Background:**

This paper describes the testicular cancer trends for incidence, survival, socio-economic status (SES) disparities and second cancer occurrence in Geneva, Switzerland, a high-risk population.

**Methods:**

We included all testicular germ-cell tumors recorded in the population-based Geneva cancer registry during the period 1970–2012. Changes in incidence trends were assessed using Joinpoint regression to calculate the annual percentage change (APC). Overall and cancer-specific survivals (OS, CSS) were estimated by Kaplan Meyer methods. To evaluate the risk of a second cancer we calculated the Standardized Incidence Ratios (SIR) using the Geneva population incidence rates.

**Results:**

The average annual testicular cancer rate was 7.32/100 000 men, with a non-significant increasing trend during the study period. The highest rates were observed among men younger than 39 years. Despite a trend toward earlier diagnosis, 14% of patients were diagnosed at a late stage. Patients with non-seminoma tumours and patients with low SES were more often diagnosed with an advanced stage. Both OS and CSS improved during the study period but with strong differences by age, stage, morphology and SES. The risk for developing a second cancer was more than doubled. This risk was particularly high for a contralateral testicular cancer, bladder cancer and pancreatic cancer.

**Conclusions:**

Overall, there was no substantial increase in the incidence of testicular cancer in Geneva in recent decades, however the prognosis has improved. The high risk of developing a second cancer, the differences in stage at diagnosis and survival by SES, require enhanced awareness and surveillance by clinicians, patients and men in general.

## Background

Testicular cancer is a rare cancer, with an annual incidence rate of 1.5 cases/100′000 men (world adjusted). In Western Caucasian populations in recent decades, there has been a sharp increase of the rate of this disease, and in Norway and Switzerland the rate went up to 12/100′000 [1]. The disease is more frequent in young men, aged less than 49 years. Hence, in 2012 in Switzerland the rate among men aged 15–39 years reached 20.9/100′000, representing the most prevalent cancer diagnosed in this age group [1].

Overall, in these countries has been observed an increase in the burden of the disease, also due to an important decrease in mortality rates following the advent of cisplatin-based chemotherapy [1, 2].

The disease has important physiological and psychological impacts on affected men and their families. Given their young age, issues of concern include not only recovery but also the consequences of both the disease and the treatment on sexuality and reproductive capacity.

There is a paucity of data regarding testicular cancer in Switzerland. The latest report from the Canton of Vaud showed one of the highest incidence rates in the world for the years 1974–1999, but with no clear upward trend since early 1990s [3–5].

The aim of this study is to provide an overall picture of testicular cancer in the high-risk canton of Geneva by studying the evolution for over 40 years of its incidence, prognosis, and occurrence of second cancers using population- based registry data.

## Methods

### Patients and data

We used data from the population-based Geneva Cancer Registry, which records information on all incident cases of malignant neoplasms occurring in the population of the canton (approximately 490′000 inhabitants) since 1970. Information collected by the registry includes patient’s sociodemographic data, tumor data, in particular on the method of detection, histology, stage, treatment in the first 6 months after diagnosis, survival, and occurrence of second tumours. Data are systematically abstracted from hospital and laboratory records by trained tumour registrars. To collect missing clinical and therapeutic data, special questionnaires are sent out regularly to the private practitioners. Death certificates are consulted systematically.

From this database, we identified 624 men resident in the canton of Geneva who were diagnosed with a primary invasive testicular cancer between 1970 and 2012. Cases with non-germinal testicular cancer (24 lymphomas, two Sertoli cell carcinomas, seven Leydig cell tumors, and one leiomyosarcoma) were excluded.

Sociodemographic variables of interest for the study were age (≤29 years, 30–39 years, 40–49 year, 50+ years), place of birth (Switzerland, Europe, Other), socio-economic status (SES) categorized in three levels based on the patient’s last occupation (low (manual employees, skilled and unskilled workers, including farmers), middle (non-manual employees and administrative staff), and high (professionals, executives, administrators, entrepreneurs)) and period of diagnosis (1970–1979, 1980–1989, 1990–1999, 2000–2012).

We considered the following variables to describe the tumour characteristics: method of detection (symptoms, fortuitous, routine check-up, autopsy or unknown); stage, categorized in four classes based on the pathologic tumor-node-metastasis (TNM) classification or, when absent, the clinical TNM classification; and morphology grouped in two classes: seminoma (International Classification of Diseases in Oncology version 10: 9060–9064 [6]) and non-seminoma (including embryonal carcinoma ICD-O 10: 9070, yolk sac tumor ICD-O 10: 9071, teratoma ICD-O 10: 9080, 9082, 9083, 9102, teratocarcinoma ICD-O 10: 9081, choriocarcinoma ICD-O 10: 9100, 9101, mixed germ-cell tumor ICD-O10: 9085).

The type of treatment received by the patients in the first 6 months after diagnosis was classified as surgery (yes vs. no), radiotherapy (yes vs. no) and chemotherapy (yes vs. no).

### Statistical methods

We calculated the annual incidence rates per 100′000 men for 11 5-year-periods. We assessed trends in incidence rates using Joinpoint regression [7].

We compared patient, tumour and treatment characteristics by stage using χ2 (homogeneity and trend tests). Unknown categories were not included in the calculation of the χ2.

Person-years at risk of developing a second primary cancer (skin non-melanoma excluded) were calculated from the date of testicular cancer diagnosis to the date of the second cancer, date of death, date of departure or 31 December 2013, whichever came first. The expected number of cancer cases was calculated by multiplying the period-age and sex-specific cancer incidence rates of the Geneva population for the period 1970–2012 by the person-years stratified in 5-year intervals. The standardized incidence ratio (SIR) was defined as the ratio between the number of observed cases and the number of expected cases. We calculated SIRs by morphology, stage, treatment and second site of malignancy. A 2-tailed 95% confidence interval (CI) of the SIR was calculated assuming a Poisson distribution of the observed numbers [8].

The patients were followed for vital status from the date of diagnosis to the date of death, the date of departure from the Canton or 31 December 2013, whichever came first. Overall and testicular cancer- specific survival (OS and CSS, respectively) were estimated using the Kaplan Meier method and stratified by age at diagnosis, period of diagnosis, socio-economic status, stage and morphology. Survival differences were tested through log rank test. For the analyses on second primary cancer and survival, one case that was discovered at autopsy has been excluded (*n* = 589). Survival analyses were performed for the whole study period as well as for the most recent years (1990–2012).

## Results

The final cohort comprised 590 patients diagnosed with testicular germ cell tumors between 1970 and 2012. Of these, 340 were diagnosed with seminoma and 250 with non-seminoma.

**Fig. 1:**
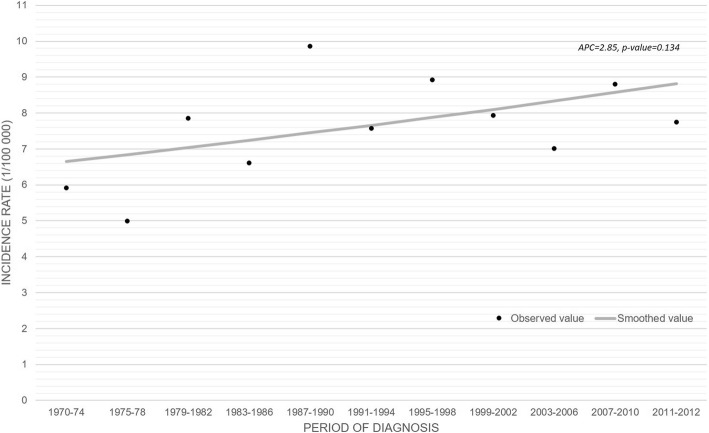
Observed and smoothed incidence of germinal testicular cancer. 1970–2012

**Fig. 2:**
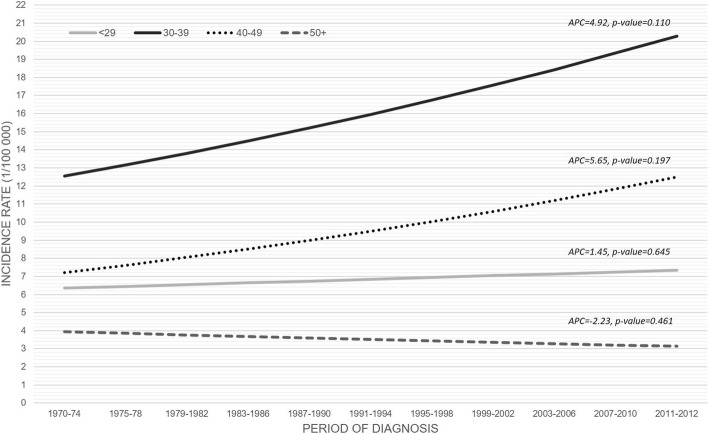
Observed and smoothed incidence of germinal testicular cancer stratified by age groups. 1970–2012

The incidence rate increased slightly during the period 1970–2012 (APC = 2.85; *p* = 0.134) but the trend was not statistically significant (Fig. 1). The trends by age group showed that the most substantial increase was observed in men aged 30–49 years (APC = 5.65, *p* = 0.197), and the highest incidence rate among patients aged 30–39 years (8.88/100′000) (Fig. 2).

Table 1 shows the distribution of patient, tumor and treatment characteristics according to stage at diagnosis. During the study period, testicular cancer was diagnosed at an earlier stage: in 1970–1979 only 54% of all cancers were diagnosed at stage I while this proportion reached 72% in the period 2000–2012 (Chi2 test: *p* = 0.038, test for trend: *p*-value = 0.016). Lower SES was found to be associated with later stage at diagnosis: 21% of low SES men were diagnosed with stage III disease compared with 13 and 9% of the medium and high SES men, respectively (Chi2 test: *p* = 0.034, test for trend: *p*-value = 0.02). Regarding the morphology of the tumour, patients with non-seminoma were diagnosed with more advanced disease compared to seminoma (22, and 7.3% diagnosed at stage III respectively, *p*-value< 0.001). Radiotherapy was administered to 59.7 and 8.4% of patients with seminomas and non-seminomas, respectively. Chemotherapy was given to 17.4 and 54.4% of patients with seminomas and non-seminomas, respectively. Patients with an early stage at diagnosis were more likely to receive radiotherapy (72% at stage I vs. 5% at stage III; *p*-value = 0.001) and less likely to receive chemotherapy (29.7% at stage I vs. 36.4% at stage III, p = < 0.001). These results were also significant when the analysis was limited to patients diagnosed in the most recent decades (1990–2013) (data not shown). Seven patients did not undergo surgery for their tumour. No association with stage at diagnosis was found for age and place of birth.

**Table 1:** Socio-demographic, tumor and treatment characteristics according to clinical TNM among 590 men with TGCT. Geneva 1970–2012

	Stage I		Stage II		Stage III		Unknown	Total		*p*-value for Chi2 heterogeneity test^a^
	N	%	N	%	N	%	N	N	%	
Period										
1970–1979	39	54.2	21	29.2	12	16.7	11	83	100	0.038
1980–1989	74	59.7	32	25.8	18	14.5	9	133	100	
1990–1999	105	70	24	16	21	14	9	159	100	
2000–2012	153	71.8	32	15	28	13.2	2	215	100	
Age group										
≤ 29	116	62	39	20.9	32	17.1	13	200	100	0.089
30–39	144	73.1	28	14.2	25	12.7	9	206	100	
40–49	69	65.1	27	25.5	10	9.4	4	110	100	
50+	42	60.9	15	21.7	12	17.4	5	74	100	
Place of birth										
Switzerland	289	68	75	17.6	61	14.4	25	450	100	0.342
Europe	61	59.8	26	25.5	15	14.7	6	108	100	
Others	21	65.6	8	25	3	9.4	0	32	100	
Social class										
High	117	73.1	28	17.5	15	9.4	11	171	100	0.034
Medium	142	67.6	40	19	28	13.3	11	221	100	
Low	81	57.4	31	22	29	20.6	6	147	100	
Unknown	31	60.7	10	19.6	7	13.7	3	51	100	
Morphology										
Seminoma	245	74.5	60	18.2	24	7.3	11	340	100	<0.001
Non-seminoma	126	50.4	49	19.9	55	22	20	250	100	
Origin of diagnosis										
Symptoms	311	65.3	92	19.3	73	15.3	16	492	100	0.736
Fortuitous	31	73.8	7	16.7	4	9.5	1	43	100	
Check	8	66.7	3	25	1	8.3	0	12	100	
Autopsy							1	1	100	
Unknown	21	72.4	7	24.1	1	3.4	13	42	100	
Radiotherapy										
Seminoma										
No	94	70.7	22	16.5	17	12.8	4	137	100	0.007
Yes	151	77.0	38	19.4	7	3.6	7	203	100	
Non-seminoma										
No	122	57.6	39	18.4	51	24.1	17	229	100	0.001
Yes	4	22.2	10	55.6	4	22.2	3	21	100	
Chemotherapy										
Seminoma										
No	230	85.2	36	13.3	4	1.5	9	270	100	< 0.001
Yes	15	25.4	24	40.7	20	33.9	2	59	100	
Non-seminoma										
No	83	88.3	7	7.5	4	4.3	16	94	100	< 0.001
Yes	43	31.6	42	30.9	51	37.5	4	136	100	

^a^missing data are not considered for the chi2 test

By the end of the study period, 106 deaths had occurred, 48 of which had testicular cancer as the underlying cause. The median follow-up was 10.9 years (range 0.06–42.4 years). Over the entire study period, the 10-year overall and cause-specific survival estimates were 88% (95% CI: 84–90) and 92% (95% CI: 89–94), respectively.

The 10-year survival trend for the whole study period, as well as survival stratified by age, SES, stage and morphology for the most recent years (1990–2012) are presented in Table 2. Both overall and cause specific survival improved significantly from the period 1970–1979 to 2000–2012 (from 65 to 94% for overall survival, *p*-value< 0.001; from 70 to 97% for cause-specific survival, *p*-value< 0.001). As the survival trend appeared to be stable from 1990 onwards, the results of survival by age, SES, stage and morphology were presented only for the more recent period.

**Table 2:** 10-year overall and cancer-specific survival after a testicular cancer diagnosis. Geneva 1970–2012

	Overall			Specific		
	% surviving	95% CI	Log-rank	% surviving	95% CI	Log-rank
All patients	87%	[84–90]		92%	[89–94]	
By period						
1970–79	65%	[54–75]	< 0.001	70%	[59–79]	< 0.001
1980–89	86%	[78–91]		93%	[86–96]	
1990–99	94%	[88–97]		96%	[91–98]	
2000–12	94%	[87–97]		97%	[93–99]	
Patients diagnosed 1990–2012	94%	[90–96]		96%	[93–98]	
By age						
≤ 29	94%	[86–98]	0.015	96%	[90–99]	0.440
30–39	97%	[91–99]		98%	[94–100]	
40–49	94%	[83–98]		94%	[83–98]	
50+	83%	[61–93]		94%	[79–99]	
By social class						
High	94%	[85–98]	0.182	98%	[91–99]	0.078
Medium	97%	[91–99]		98%	[94–100]	
Low	88%	[77–94]		90%	[79–95]	
Unknown	91%	[65–98]		97%	[81–1.00]	
By stage						
I	96%	[92–98]	< 0.001	99%	[96–100]	< 0.001
II	93%	[74–98]		95%	[72–99]	
III	81%	[67–90]		81%	[67–90]	
Unknown	–	–		100%	–	
By morphology						
Seminoma	96%	[91–98]	0.014	98%	[94–100]	< 0.001
Non-seminoma	90%	[83–95]		93%	[87–96]	

Patients of low SES presented a lower specific survival compared with those from high social class (90, 95% CI: 79–95, vs. 98, 95% CI: 91–99) but the difference was not statistically significant (*p*-value = 0.078) for the period 1990–2012. It is however worth noting that when considering the whole period (1970–2012), SES was significantly associated with 10-year overall and specific survival SES (data not shown). We found a large difference in survival by stage at diagnosis, particularly for cancer-specific survival wherein men with stage I disease had a 10-year survival of 98% (95% CI: 96–100) while for men diagnosed with at stage III it was 81% (95% CI: 67–90) (*p*-value for log rank test< 0.001). Survival was higher for seminomas than non-seminomas for both overall and testicular cancer death (*p*-value for log-rank test< 0.001). Regarding morphology subtypes, patients with seminoma presented significantly higher survival rate (10-year overall survival: 96, 95%CI: 91–98; 10-year specific survival: 99, 95% CI: 94–100) compared to non-seminoma tumours.

Among the 590 patients diagnosed with testicular cancer, 70 had had a second primary malignancy by the end of the follow-up period compared with 34.7 expected (SIR 2.02, 95% CI: 1.6–2.5, *p*-value = < 0.001) (Table 3). The risk for contralateral cancer of the testis was particularly high (SIR: 20.7, 95% CI: 11.9–33.7, *p*-value = < 0.001). The risk for pancreatic and bladder cancers was also increased (SIR 3.76, 95% CI: 0.8–11, *p*-value = 0.045 and SIR 3.98. 95% CI: 1.5–8.6, *p*-value = 0.005, respectively). The risk of pancreatic cancer was especially high for patients diagnosed with non-seminoma (SIR 9.8, 95% CI: 1.1–34, p- value = 0.018) and for those who had chemotherapy (SIR 13.0, 95% CI: 1.5–48.1, *p*-value = 0.011). The risk of bladder cancer was higher among patients with seminoma (SIR 4.4, 95% CI: 1.4–10.2, *p*-value = 0.006) and those treated with radiotherapy (SIR 5.1, 95% CI: 1.6–11.8, *p*-value = 0.004) (data not shown).

**Table 3:** Standardized Incidence Ratios (SIR) for second primary cancer after a testicular cancer diagnosis according to site. Geneva 1970–2012

Localisation of the second cancer	Observed	Expected	SIR	95% CI	*p*-value
Controlateral testis	16	0.77	20.66	[11.9–33.7]	< 0.001
Pancreas	3	0.8	3.76	[0.8–11]	0.047
Bladder	6	1.51	3.98	[1.5–8.6]	0.005
Prostate	10	7.47	1.34	[0.6–2.5]	0.221
Stomach	2	0.93	2.15	[0.2–7.8]	0.239
Lung	6	5.4	1.2	[0.4–2.4]	0.385
All tumors (skin non-melanoma excluded)	70	34.73	2.02	[1.6–2.5]	< 0.001

## Discussion

This study shows that despite the high and increasing rate of testicular cancer in Switzerland overall, in Geneva the rate did not increase significantly between 1970 and 2012. However, there has been a clear trend towards earlier diagnosis and a significant improvement in survival in Geneva during this period, although differences by morphology and, to a lesser extent, by SES persist. The risk of developing a second cancer, particularly a contralateral testicular cancer, bladder or pancreatic cancer, is very high in these patients compared with the Geneva population.

A majority of Western countries have reported an increase in testicular cancer rates in recent decades [9, 10]. In Switzerland an increasing trend of testicular cancer has been observed over a period of 35 years with a growth of 1.4% every 2 years (95% CI: 0.7–2.0; *p* < 0.001). The increasing trend seems to be mainly driven, however, by cantons in the German speaking region which have higher incidence rates than those observed in the French speaking region, to which Geneva belongs [11]. In particular, the rates for the period 2011-2015was 12.1 (95% CI: 11.5–12.8) for the German speaking region and 8.9 (95% CI: 8.2—9.7) for the French speaking region. During the period 1970–2012, we observed only a not statistically significant slightly increasing trend, which was similar to that observed in the French-speaking canton of Vaud [4]. The authors of that study did not find an upward trend in testicular cancer rates during the period 1974–1999; they related their finding to the high testicular cancer rate already reached in the early 1990s [5].

Our results confirm that survival for testicular cancer has improved significantly since the 1970s, with 10- year cancer-specific survival now reaching 97%. This improvement can be ascribed to improvements in treatment, particularly with the advent of cisplatin and well defined management recommendations for the disease [12–14]. Survival rates are clearly associated with stage at diagnosis and morphology. In particular, patients in our study who were diagnosed at an advanced stage or with a non-seminoma cancer had a significantly worse survival, consistent with other studies [15].

Results about SES were not statistically significant and must be interpreted with caution. However, this study suggests that men with a low SES experience worse overall and cancer-specific survival in Geneva, despite obligatory health insurance which allows almost uniform access to healthcare and treatments [16]. SES inequalities observed in testicular cancer studies in England and Wales were attributed to differences in stage at diagnosis and access to treatment [17, 18]. In our study, men of low SES were more often diagnosed with an advanced stage, which could partially explain their lower survival and suggest delay in disease detection. Given the general consensus that routine screening of asymptomatic men, whether with palpation or biomarkers, is ineffective [12–14, 19], and that over 80% of our patients were diagnosed based on symptoms, it is important that both patients and clinicians have a high index of suspicion for this disease to avoid delay in diagnosis [14].

Because of their young age at diagnosis and the improving prognosis, men diagnosed with testicular cancer are at increased risk of developing a second primary cancer during their lifetime. Compared with the Geneva population, our study population showed a doubling of their risk of a second cancer, especially of the contralateral testicular, pancreas or bladder. This is consistent with other reports. The increased risk of second primary cancers has been associated with the use of adjuvant therapies [20, 21]. We found an association between the use of radiotherapy and an excess risk of secondary bladder cancer, an infra-diaphragmatic site exposed to the radiotherapy field and between an excess of pancreatic cancer and treatment with chemotherapy, particularly in non-seminoma cancers. These findings align with those of other studies [21–24]. That said, the combined effect of both radiotherapy and chemotherapy on the risk of pancreatic cancer remains unclear. A previous study evaluating the risk of pancreatic cancer after treatment for Hodgkin’s lymphoma suggested that patients who had both radiotherapy and > = 6 cycles of alkylating agents chemotherapy presented the highest risk [25]. Further studies should investigate if the same effect is observed for testicular cancers.

The risk of a second primary cancer for contralateral testicular cancer was extremely high and of the same order of magnitude as seen in other studies [21, 26–29]. Cryptorchidism, environmental exposures, epigenetic aberrations and genetic susceptibility are the suggested etiologic mechanisms for the development of this cancer [30]. The risk factors for a first testicular cancer can predispose for a second such cancer. However, in our study the risk of a second testicular cancer in patients who did not receive adjuvant therapies was the same as the risk for primary testicular cancer in the general population. This supports the theory that chemotherapy is a risk factor for the development of a second testicular cancer, contrasting the results of a study that found a reduced risk after chemotherapy with alkylating agents [31].

One limitation of our study is the relatively small number of cancer cases due to the rarity of testicular cancer and the small size of the population under study. Nevertheless, we included all of the 590 testicular cancer cases that occurred in the Geneva population over a 40-year period.

Another potential limitation is the possible lack of completeness of case ascertainment or lack of completeness of follow-up and treatment data. However, the accuracy of the Geneva cancer registry is rather high [32] in general and again as demonstrated in this study by the fact that only 1 case was discovered after death. Furthermore, the median time of follow-up was 11 years, cumulating in 7733 person-years of observation. Another marker of accuracy of the data is that all included cases were morphologically defined. Given the above, the findings are definitively generalizable to the overall patient population of Geneva, while caution should be used when trying to generalize them to other settings.

## Conclusions

In contrast to the overall situation in Switzerland, there has been no significant increase in the incidence of testicular cancer in Geneva since 1970. At the same time, survival rates have been steadily improving. However, given the young age of patients affected, the very high risk of developing a second cancer, and the existence of strong inequalities in terms of stage at diagnosis and survival, heightened awareness of testicular cancer and its risks is vital, among both patients and clinicians, to promote early diagnosis and active surveillance of men diagnosed with this cancer.

## Data Availability

The datasets used and/or analysed during the current study are available from the corresponding author on reasonable request. In compliance with data protection regulations, data are stored at the Geneva Cancer Registry, Geneva, Switzerland.

## Abbreviations

APC: Annual Percent Change
CI: Confidence Interval
CSS: Cause-Specific Survival
ICDO: International Classification of Diseases in Oncology
OS: Overall Survival
SES: Socio Economic Status
SIR: Standardised Incidence Ratio
TNM: Tumor-Node-Metastasis
